# Extracts from cultures of *Pseudomonas fluorescens* induce defensive patterns of gene expression and enzyme activity while depressing visible injury and reactive oxygen species in *Arabidopsis thaliana* challenged with pathogenic *Pseudomonas syringae*

**DOI:** 10.1093/aobpla/plz049

**Published:** 2019-07-29

**Authors:** H Martin-Rivilla, A Garcia-Villaraco, B Ramos-Solano, F J Gutierrez-Mañero, J A Lucas

**Affiliations:** Plant Physiology, Pharmaceutical and Health Sciences Department, Faculty of Pharmacy, Universidad San Pablo-CEU Universities, Boadilla del Monte, Spain

**Keywords:** ISR, metabolic elicitors, oxidative stress, *Pseudomonas fluorescens*, salicylic acid (SA) and jasmonic acid/ethylene (JA/ET) signalling pathway

## Abstract

We evaluated the ability of metabolic elicitors extracted from *Pseudomonas fluorescens* N21.4 to induce systemic resistance (ISR) in *Arabidopsis thaliana* against the pathogen *Pseudomonas syringae* DC3000. Metabolic elicitors were obtained from bacteria-free culture medium with *n*-hexane, ethyl acetate and *n*-butanol in three consecutive extractions. Each extract showed plant protection activity. The *n*-hexane fraction was the most effective and was used to study the signal transduction pathways involved by evaluating expression of marker genes of the salicylic acid (SA) signalling pathway (*NPR1*, *PR1*, *ICS* and *PR2*) and the jasmonic acid/ethylene (JA/ET) signalling pathway (*PDF1*, *MYC2*, *LOX2* and *PR3*). In addition, the level of oxidative stress was tested by determining the activity of enzymes related to the ascorbate-glutathione cycle. *N*-hexane extracts stimulated both pathways based on overexpression of *ICS*, *PR1*, *PR2*, *PDF1* and *LOX2* genes. In addition, activity of the pathogenesis-related proteins glucanase (PR2) and chitinase (PR3), lipoxygenase and polyphenol oxidase was enhanced together with an increased capacity to remove reactive oxygen species (ROS). This was associated with less oxidative stress as indicated by a decrease in malondialdehyde (MDA), suggesting a causative link between defensive metabolism against *P. syringae* and ROS scavenging.

## Teaser

With this study, and by using three different organic solvents, we extracted some molecules derived from the metabolism of the beneficial strain *Pseudomonas fluorescens* N21.4, which protected *Arabidopsis thaliana* plants from attack by the pathogen *Pseudomonas syringae* DC3000. The molecules extracted using the organic solvent *n*-hexane were the most efficient at protection. They were also capable of activating the two defensive pathways of the plant immune system and all the enzymes responsible for reducing the oxidative stress that plants experience when they are subjected to some kind of attack.

## Introduction

Agricultural systems impose a range of abiotic and biotic stresses on crop plants that lower their productivity (García-Cristobal *et al.* 2015), thus compromising food supplies worldwide (Pechanova and Pechan 2015; Miller *et al.* 2017).

Due to its significance, interactions between plants and pathogenic organisms have been studied intensively with a view to providing sustainable solutions for crop diseases, to enhance food safety by improving food quality and crop yields and to understand how plants cope with biotic stress (Silva *et al.* 2018).

The rapid generation of reactive oxygen species (ROS, such as O_2_^•−^, H_2_O_2_, and ^•^OH) represents a common plant response to pathogen attack (Noctor *et al.* 2014; Xia *et al.* 2015) and therefore represents a node from which many signalling events are generated. A rise in ROS production leads to oxidative stress (Gill and Tuteja 2010) mainly by provoking oxidative modification of vital biomolecules including membrane lipids, cellular amino acids, proteins and DNA (Gill and Tuteja 2010; Anjum *et al.* 2012). The outcomes include cell death and the arrest of plant growth and development. To maintain optimal levels of ROS, plants possess a sophisticated regulatory system consisting of enzymatic antioxidants (superoxide dismutase, SOD; catalase, CAT; guaiacol peroxidase, GPX; ascorbate peroxidase, APX; monodehydroascorbate reductase, MDHAR; dehydroascorbate reductase, DHAR; glutathione reductase, GR) and non-enzymatic antioxidants (ascorbate, ASC; glutathione, GSH; carotenoids; tocopherols; phenolics compounds).

Colonization of plant roots by PGPR (plant growth-promoting rhizobacteria) improves plant health by stimulating its immune system to decrease oxidative stress through improving ROS scavenging (Lucas *et al.* 2013, 2014; García-Cristobal *et al.* 2015). This phenomenon is known as induced systemic resistance (ISR) and involves the induction of resistance not only locally at the site of infection, but also systemically. Induced systemic resistance has been primarily described as a response induced by PGPR (Pieterse *et al.* 2000), but it can also be induced by metabolic elicitors such as antibiotics, surfactants or other chemicals (Gozzo and Faoro 2013). The elicitation of defensive metabolism by PGPR or elicitors leads to a physiological situation in the plant called priming (Conrath 2011). In this situation, plants show faster and/or stronger activation of defence responses when subsequently challenged by pathogen (Conrath *et al.* 2006).

Despite the many studies of PGPR triggering ISR, few have focused on the molecular elicitors produced by these bacteria. However, metabolites from various bacterial genera: *Klebsiella* (Park *et al.* 2009), *Ochrobactrum* (Sumayo *et al.* 2013), *Pseudomonas* (Ongena *et al.* 2005) and *Bacillus* (Huang *et al.* 2012) have been recognized as ISR metabolic elicitors, with those from *Bacillus* being the most studied, although it is well-known that *Pseudomonas* spp. are possibly the most important producers of compounds triggering plant immune responses (Durrant and Dong 2004; Choudhary *et al.* 2007). Interest in PGPR and their elicitors is heightened by their potential for developing a sustainable agriculture without pesticides or agrochemicals (Wu *et al.* 2018).

After a PGPR or their metabolic elicitors are sensed by a plant, salicylic acid (SA), jasmonic acid (JA) or ethylene (ET) signalling pathways are activated to trigger plant resistance (Wu *et al.* 2018). In the case of ISR, the response depends on JA and ET signalling and also requires NPR1 (non-expressor of pathogenesis-related protein 1) (Pieterse and Van Loon 2004, 2007). The JA signalling pathway has two branches controlled by the transcription factor *MYC2* and ethylene response factor (ERF). The ERF branch of the JA pathway is associated to enhance resistance to necrotrophic pathogens and one of the marker genes of this branch is plant defensin 1 (*PDF1*) (Berrocal-Lobo *et al.* 2002; Lorenzo *et al.* 2003).

The aim of the present work was (i) to obtain extracts containing bacterial metabolic elicitors able to trigger protection against pathogens in the model plant *Arabidopsis thaliana* and (ii) to determine the transduction signal pathways involved in this protection. Three organic fractions were obtained from the culture media of a strain of *Pseudomonas fluorescens* (N21.4), a gram-negative bacilli isolated from the rhizosphere of *Nicotiana glauca* (Ramos-Solano *et al.* 2010b). This bacterium is known to trigger defensive metabolism in *Solanum lycopersicum* and *A. thaliana* (Domenech *et al.* 2007), to increase isoflavone content in *Glycine max* (Ramos-Solano *et al.* 2010a), to promote fruit production in *Rubus* sp. (Ramos-Solano *et al.* 2014) and to improve fruit quality of *Rubus* sp. by modifying flavonoid metabolism (Garcia-Seco *et al.* 2015). We also wished to evaluate the ability of the extracts to trigger plant defence against pathovar DC3000, a pathogenic strain of *P. syringae*. The most effective of the three fractions was then used to study the signal transduction pathway. To reach these objectives differential gene expression of marker genes from the SA and JA/ET pathways was analysed as well as enzymes involved in ROS scavenging system and proteins involved in induction systemic resistance, all in the context of the overall oxidative status of the plant.

## Materials and Methods

### Bacterial pathogen, model plant used and metabolic elicitors extraction

*Pseudomonas syringae* (DC3000) was used as the pathogen in the experiments for challenge inoculation. This strain causes bacterial speck on the model plant *A. thaliana* and is used to study the model system for plant–pathogen interactions (van Loon *et al.* 1998). The pathogen was grown for 24 h in 100 mL of nutrient broth (Conda; gelatin peptone 5 g L^−1^ and beef extract 3 g L^−1^) in a 250-mL Erlenmeyer flask on a shaker (125 rpm) at 28 °C. The culture was then centrifuged (350 × *g* for 10 min), washed with sterile water and pellet was suspended in sterile sufficient 10 mM MgSO_4_ to achieve 10^8^ cfu mL^−1^. The enumeration and calculations were carried out following the ‘drop method’ (Hoben and SomesegAran 1982).

*Arabidopsis thaliana* Columbia ecotype was used. Seedlings were incubated in a culture chamber (Sanyo MLR-350H) with a 9 h light (350 μE s^−1^·m^−2^ at 24 °C) and 15 h dark cycle (20 °C) at 70 % relative humidity.

Metabolic elicitors from *P. fluorescens* (N21.4) were obtained according to Sumayo *et al.* (2013) using three separate solvents. The bacterium was first grown in nutrient broth (Conda) on a rotary shaker (180 rpm) at 28 °C for 24 h. Cells were separated by centrifugation at 8000 × *g* for 15 min, and 500 mL of the supernatant filtered (0.2 μm) and extracted sequentially in *n*-hexane, ethyl acetate and *n*-butanol to obtain the metabolic elicitors. The dry residues from each fraction were dissolved in 25 mL 10 % dimethylsulfoxide (DMSO).

### Screening for the most effective determinant fraction to trigger systemic resistance

An ISR assay on *A. thaliana* plants was used to evaluate the ability of three fractions from *P. fluorescens* (N21.4) to trigger plant protection. The following five treatments were involved: (i) metabolic elicitors in the *n*-hexane fraction, (ii) metabolic elicitors in the ethyl acetate fraction, (iii) metabolic elicitors in the *n*-butanol fraction, (iv) N21.4 (positive control) and (v) untreated plants (negative control). An additional control with 10 % DSMO was included to ensure that the effects were due to metabolic elicitors and not to the DMSO. All were pathogen challenged.

*Arabidopsis thaliana* seeds (not previously sterilized) were germinated in quartz sand for 1 week and then transplanted individually to 12-well plastic plates (5 mL) filled with peat. Each treatment comprised three plates, each plate constituting a replicate. One week after transplanting, treatments were delivered to seedlings by drenching in the soil with 20 μL of elicitors per well. The positive control was inoculated with 1 mL of 10^8^ cfu mL^−1^ of N21.4 culture, grown for 24 h in sterile nutrient broth (Conda) while negative controls were treated with 1 mL of sterile nutrient broth (Conda) or 20 μL of 10 % DMSO.

Three days later, plates were placed in a humidity chamber to ensure stomata opening, and the next day challenged with pathogen *P. syringae* DC3000. The plants were inoculated by placing a 5 μL drop of 10^8^ cfu mL^−1^ suspension on each leaf (Sumayo *et al.* 2013). Seventy-two hours after pathogen inoculation, the disease index was determined as the ratio of the number of leaves with disease symptoms to the total number of leaves (Ryu *et al.* 2004). Results were relativized using the negative control as a 0 % of protection.

### Study of the signal transduction pathway of the most effective fraction

The *n*-hexane fraction was the most effective against pathogen infection. This was used to study signal transduction pathways based on genes overexpressed in response to this fraction during the systemic resistance assay. The genes involved are detailed below. The experimental set-up included two treatments: (i) metabolic elicitors in the *n*-hexane fraction, and (ii) pathogen-only control (negative control). Twenty-one plants per treatment were used; plants were arranged on three replicates, with seven plants each.

Seeds were germinated in quartz sand for 1 week. One-week-old seedlings were transplanted individually to 100 mL pots filled with 3:1 (vol/vol) peat/sand mixture (60 g per pot). Plants were watered with 5 mL of tap water twice a week, and with 5 mL of ½ Hoagland solution per plant once a week. Four-week-old seedlings were treated with 50 μL of *n*-hexane fraction, and negative control with 50 μL of the *n*-hexane fraction from sterile nutrient broth. Four days later, plants were pathogen challenged.

One day before the pathogen inoculation, plants were placed in a humidity chamber to ensure the stomatal opening needed for the disease to establish. Pathogen inoculation was carried out by spraying the plants with 150 mL of a suspension of 10^8^ cfu mL^−1^; non-pathogen controls were mock inoculated with sterile nutrient broth (Conda). Seven plants per treatment were collected 6, 12 and 24 h after pathogen challenge (hapc), powdered in liquid nitrogen and stored at −80 °C until gene expression analysis by qPCR and enzymatic activities analysis.

The genes analysed were *NPR1* (non-expressor of pathogenesis-related gene 1), *PR1* (pathogenesis-related gene 1), *PR2* and *ICS* (isochorismate synthase 1) as markers of the SA signalling pathway (Betsuyaku *et al.* 2017; Ding *et al.* 2018; Silva *et al.* 2018); *PDF1*, *LOX2* (lipoxygenase 2), *PR3* and the transcriptional factor *MYC2* as markers of the JA/ET signalling pathway (Caarls *et al.* 2015).

### RNA extraction and RT-qPCR analysis

Total RNA was isolated from each replicate with PureLink RNA Micro Kit (Invitrogen), DNAase treatment included. RNA purity was confirmed using Nanodrop™. A reverse transcription was performed followed by qPCR. Reverse transcription was performed using iScript tm cDNA Synthesis Kit (Bio-Rad). All reverse transcription were carried out using a GeneAmp PCR System 2700 (Applied Biosystems): 5 min 25 °C, 30 min 42 °C, 5 min 85 °C, and hold at 4 °C. The amplification was realized with a MiniOpticon Real Time PCR System (Bio-Rad): 3 min at 95 °C and then 39 cycles consisting of 15 s at 95 °C, 30 s at 55 °C and 30 s at 72 °C, followed by a melting curve to check the results. To describe the level of expression in the analysis, cycle threshold (Ct) was used. Standard curves were calculated for each gene, and the efficiency values ranged between 90 and 110 %. *Sand* gen (AT2G28390) was used as a reference gen. Primers used appear in **Supporting Information—Table S1**. Results for gene expression were expressed as differential expression by the 2^−ΔΔCt^ method.

### Pathogenesis-related proteins and systemic resistance proteins activities

Enzymatic activities of resistance proteins glucanase (PR2), chitinase (PR3), lipoxygenase, cellulase and polyphenol oxidase were assessed. Before assessing enzymatic activities, soluble proteins were extracted from the plant powder by resuspending 100 mg in 1 mL of potassium phosphate buffer 0.1 M pH 7 containing 2 mM phenylmethylsulfonyl fluoride (PMSF). These were sonicated 10 min and then centrifuged for 10 min at 14 000 rpm. The supernatant was divided into aliquots, frozen in liquid nitrogen and stored at −80 °C for further analysis. All above operations were carried out at 0–4 °C.

To measure the amount of total protein from plant extract, 250 µL of Bradford reagent, 50 µL of sample and BSA dilutions were pipetted into each well of 96-well plates, incubated for 30 min at room temperature and measured using a plate reader (MB-580 Heales) at absorbance of 595 nm. A calibration curve was constructed from commercial BSA dilutions expressed in milligrams. The units of protein were expressed as mg µL^−1^.

Glucanase (EC 3.2.1.6), cellulase (EC 3.2.1.4) and chitinase (EC 3.2.1.14) activities were measured as described by Lee *et al.* (2008). Calibration curves were made with glucose (for glucanase and cellulase) and *N*-acetyl glucosamine (for chitinase) in acetate buffer with concentrations between 0.1 and 1 mg mL^−1^ for glucanase and cellulase, and between 0.01 and 0.1 mg mL^−1^ for chitinase. Data were expressed as µmol mg protein^−1^ min^−1^.

Lipoxygenase (EC 1.13.11) activity was measured as described by Ali *et al.* (2005). Extinction coefficient of 25 mM^−1^ cm^−1^ was used to calculate activity. Data were expressed as µmol mg protein^−1^ min^−1^.

Polyphenol oxidase (1.14.18.1) activity was measured as described by Nawrocka *et al.* (2018). Extinction coefficient of 2.72 mM^−1^ cm^−1^ was used to calculate activity. Data were expressed as µmol mg protein^−1^ min^−1^.

In all assays, the blank consisted on the components of the reaction mixture except for the enzyme extract, which was replaced by an equal volume of the assay buffer.

### Enzymatic activities related to oxidative stress

Enzyme activities related of APX (EC 1.11.1.11), SOD (EC 1.15.1.1), GR (EC 1.6.4.2), GPX (EC 1.11.1.7), CAT (EC 1.11.1.6), MDHAR (EC 1.6.5.4) and DHAR (EC 1.8.5.1) were measured spectrophotometrically and expressed as µmol mg protein^−1^ min^−1^.

Ascorbate peroxidase was measured by the method of Garcia-Limones *et al.* (2002). Oxidation of ASC was determined by the decrease in A_290_. An extinction coefficient of 2.8 mM^−1^ cm^−1^ was used to calculate activity.

Superoxide dismutase activity was determined following the specifications of the SOD activity detection kit (SOD Assay Kit-WST, Sigma-Aldrich). With this method, the rate of the reduction with O_2_ is linearly related to xanthine oxidase (XO) activity and inhibited by SOD. Inhibition activity of SOD was determined colourimetrically and expressed as % inhibition mg protein^−1^.

Glutathione reductase was measured by the method of Garcia-Limones *et al.* (2002). Oxidation of NADPH was determined by the increase in A_340_. Extinction coefficient of 6.2 mM^−1^ cm^−1^ was used to calculate activity.

Guaiacol peroxidase was measured by the method of Garcia-Limones *et al.* (2002). Oxidation of guaiacol was determined by the increase in A_470_ using an extinction coefficient of 26.6 mM^−1^ cm^−1^ to calculate activity.

Catalase was measured by the method of Garcia-Limones *et al.* (2002). The decrease in A_240_ produced by H_2_O_2_ breakdown was recorded and an extinction coefficient of 36 mM^−1^ cm^−1^ used to calculate activity.

Monodehydroascorbate reductase activity was measured by the method of Xu *et al.* (2008). Reduction of monodehydroascorbate was determined by the decrease in A_340_ using an extinction coefficient of 6.22 mM^−1^ cm^−1^ to calculate activity.

Dehydroascorbate reductase activity was measured as described by Xu *et al.* (2008) at 265 nm. Reduction of dehrydroascorbate was determined by the decrease in A_265_ using an extinction coefficient of 14 mM^−1^ cm^−1^ to calculate activity.

In all assays, the blank consisted on the components of the reaction mixture except from the enzyme extract, which was replaced by an equal volume of the assay buffer. In the case of the GR assay, an additional blank without oxidized GSH was included to account for the presence in the extracts of other enzyme activities able to oxidize NADPH.

### Oxidative status of the plant: malondialdehyde concentration

The malondialdehyde (MDA) content was determined by the method of Hu *et al.* (2016) with modifications. Briefly, 0.25 g of powder was mixed with 2 mL of reaction solution containing 0.5 % (vol/vol) thiobarbituric acid (TBA) and 20 % (vol/vol) trichloroacetic acid (TCA). The mixture was heated at 95 °C for 30 min, then quickly cooled to room temperature, treated to eliminate air bubbles and centrifuged at 6000 × *g* for 20 min. Then, absorbance of the supernatant was determined by a spectrophotometer at 532 and 600 nm. The MDA content was calculated using the formula: MDA (nmol/FW) = [(OD532 − OD600)]/(ε·FW), where FW is the fresh weight in grams and ε the extinction coefficient (155 mM^−1^ cm^−1^).

### Statistical analysis

One-way ANOVA was used to check the statistical differences in all data obtained in the experiments carried out. Prior to ANOVA, analysis of homoscedasticity and normality of the variance were checked with Statgraphics plus 5.1 for Windows and found to meet the requirements for analysis. When significant differences appeared (*P* < 0.05) a Fisher test was used (Sokal and Rohlf 1980).

## Results

### Capacity of the three metabolic elicitors fractions to trigger systemic resistance

**Figure 1.**
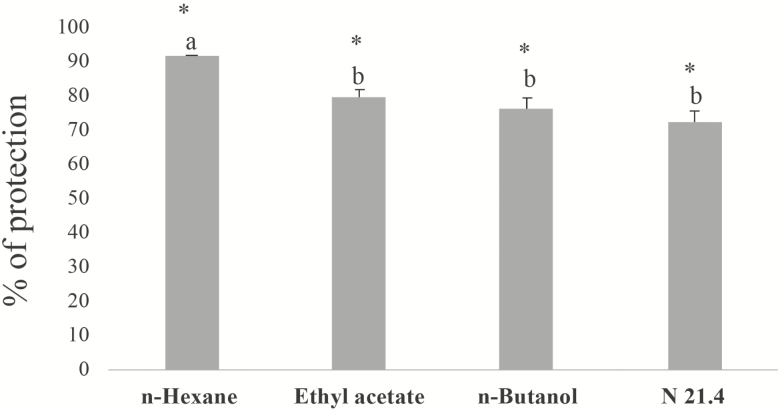
The extent of protection against the pathogen *Pseudomonas syringae* DC3000 to seedlings of *Arabidopsis thaliana* by extracts from culture media supporting *Pseudomonas fluorescens* (N21.4). Three solvent fractions (*n*-hexane, ethyl acetate, *n*-butanol) were tested and compared. Percentage of protection was based on the number of leaves with disease symptoms compared to the total of leaves (*n* = 12 seedlings). Data were relativized to control (i.e. seedlings inoculated only with pathogen), which was considered as 0 % protection. Asterisks represent statistically significant differences (*P* < 0.05) with regard to negative control. Letters represent statistically significant differences between the four different treatments.

Each of the organic fractions from culture media containing *P. fluorescens* (N21.4) and the N21.4 strain itself were able to trigger defence mechanisms in *Arabidopsis* seedlings and to improve their capacity to resist the pathogenic effects of *P. syringae* (DC3000) (Fig. 1). The *n*-hexane fraction gave the highest protection percentage (91 %) and was chosen to study the signal transduction pathway involved in protection by evaluating the differential gene expression (fold change) of selected marker genes. Negative controls treated with DMSO or sterile nutrient broth had no effect.

### Study of the signal transduction pathway of the most effective fraction

**Figure 2.**
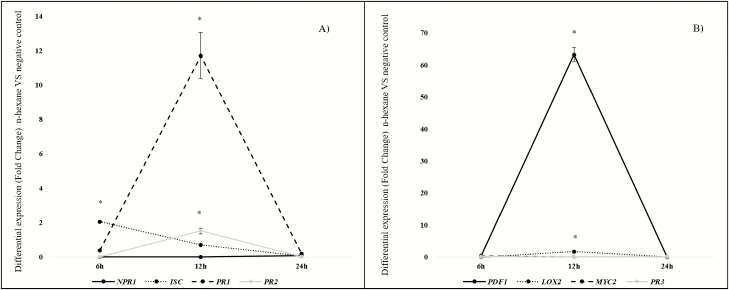
Differential expression (fold change) of SA pathway and JA/ET pathway marker genes by seedlings of *Arabidopsis thaliana* treated with *n*-hexane extract from culture media supporting *Pseudomonas fluorescens* N21.4. The results compare treatments against a negative control 6, 12 and 24 h after challenging with *Pseudomonas syringae* DC3000; (A) *NPR1*, *ICS*, *PR1* and *PR2* genes (as SA signalling pathway markers), (B) *PDF1*, *LOX2*, *MYC2* and *PR3* (as JA/ET signalling pathway markers). Asterisks represent statistically significant differences (*P* < 0.05) within each sampling time (6, 12 and 24 h; *n* = 7).

Effects of the *n*-hexane fraction on SA pathway and the JA/ET pathway marker genes are shown in Fig. 2. Figure 2A shows the SA pathway marker genes. Six hours after pathogen challenge (6 hapc) only *ICS* showed significantly higher expression (2.04), decreasing to zero values 12 and 24 hapc. *PR1* and *PR2* showed maximum differential expression at 12 hapc (11.7), although *PR2* expression was 10 times lower than for *PR1* (1.51). None of the genes showed differential expression 24 hapc.

Figure 2B shows the JA/ET pathway marker genes. Only *PDF1* (63.2) and *LOX2* (1.71) showed significant differences in gene expression. These were evident 12 hapc, with *PDF1* values being 60 times higher than *LOX2*. There was no differential gene expression 6 and 24 hapc. Negative control treated with 50 μL of *n*-hexane fraction from sterile nutrient broth had no effect on differential gene expression.

### Pathogenesis-related proteins and systemic resistance proteins activities

**Figure 3.**
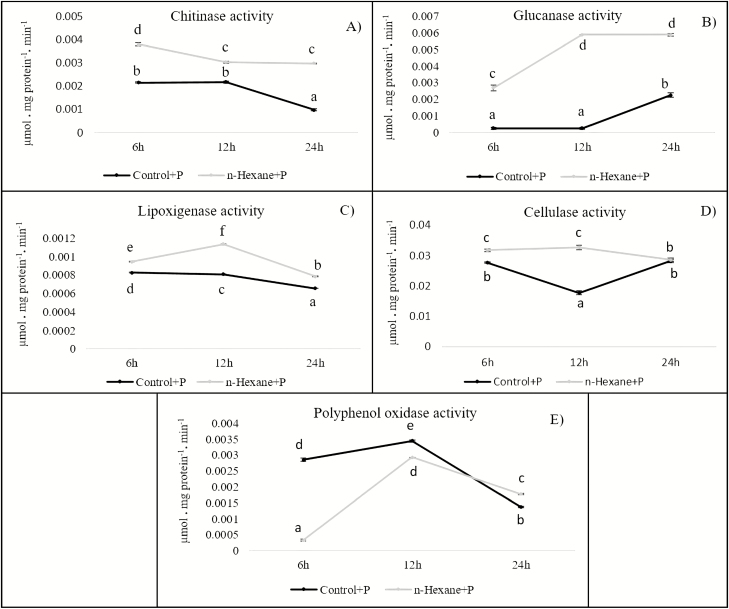
Pathogenesis-related proteins and ISR proteins activities in plants of *Arabidopsis thaliana* inoculated with the pathogenic *Pseudomonas syringae* DC3000 (Control + P) and treated with elicitor extract with *n*-hexane (*n*-hexane + P) taken from culture media supporting *Pseudomonas fluorescens* (N21.4). Activities were measured 6, 12 and 24 hapc. (A) Chitinase (PR3); (B) glucanase (PR2); (C) lipoxygenase; (D) cellulase and (E) polyphenol oxidase. Different letters indicate significant differences (*P* < 0.05) between treatments in each sampling time.

Activity of the pathogenesis-related proteins (PRs) glucanase (PR2) and chitinase (PR3) and the proteins related to systemic resistance against pathogen lipoxygenase, cellulase and polyphenol oxidase were evaluated (Fig. 3). The *n*-hexane fraction promoted the activity of all these enzymes. The increases were statistically significant at most sampling times for chitinase (PR3; Fig. 3A) glucanase (PR2; Fig. 3B) and lipoxygenase (Fig. 3C). Cellulase (Fig. 3D) showed significant differences 6 and 12 hapc, and polyphenol oxidase (Fig. 3E) 24 hapc.

### Enzymatic activities related to oxidative stress

**Figure 4.**
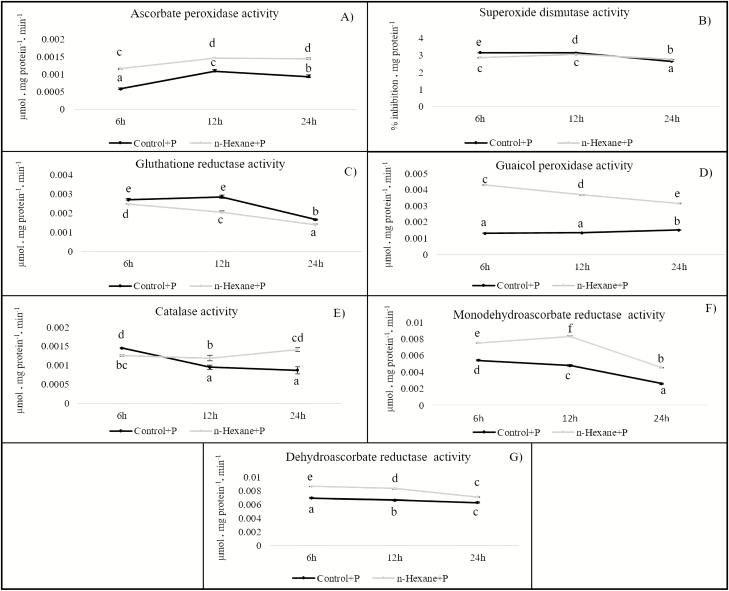
Enzyme activities related to oxidative stress in plants of *Arabidopsis thaliana* inoculated with the pathogenic *Pseudomonas syringae* DC3000 (Control + P) and treated with elicitor-containing *n*-hexane extract taken from culture media supporting *Pseudomonas fluorescens* (N21.4) (*n*-hexane + P). Enzyme assays were made 6, 12 and 24 hapc. (A) APX; (B) SOD; (C) GR; (D) GPX; (E) CAT; (F) MDHAR; (G) DHAR. Different letters indicate significant differences (*P* < 0.05) between treatments in each sampling time.

Except for glutathione reductase activity (GR; Fig. 4E), *n*-hexane fraction elicitors increased activity levels compared to controls. The differences were statistically significant at all three sampling times for APX (Fig. 4A) and GPX (Fig. 4D). Increases in CAT (Fig. 4E) and MDHAR (Fig. 4F) activity were significant 12 and 24 hapc. Superoxide dismutase activity (SOD; Fig. 4B) was promoted 6 and 24 hapc and DHAR (Fig. 4G) 12 hapc.

### Oxidative status of the plant: MDA concentration

**Figure 5.**
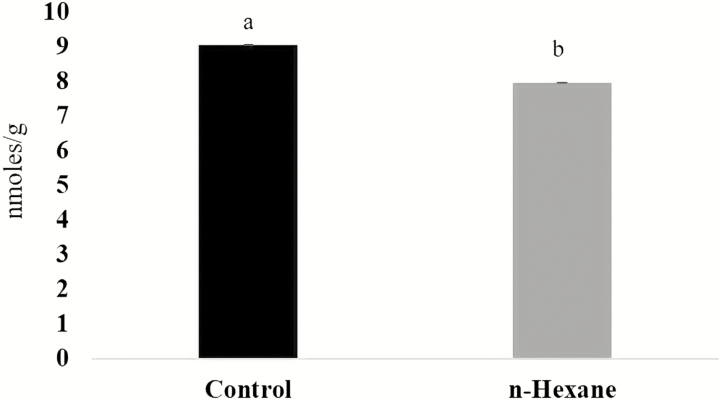
Malondialdehyde concentrations in plants of *Arabidopsis thaliana* inoculated with the pathogenic *Pseudomonas syringae* DC3000 and after treatment with *n*-hexane extracts of culture media supporting *Pseudomonas fluorescens* (N21.4) compared to controls. Different letters indicate significant differences (*P* < 0.05) between treatments.

Malondialdehyde, a marker of oxidative stress was measured 12 hapc (Fig. 5). At this time, MDA concentrations were markedly depressed by *n*-hexane extract.

## Discussion

The capacity of PGPR to enhance plant defence of biotic and abiotic stresses has been demonstrated many times in the past (e.g. García-Cristobal *et al.* 2015; Beris *et al.* 2018; Kumar *et al.* 2018). However, effects of elicitors produced by these PGPRs have been less studied. These substances have been reported to be either structural molecules, such as flagellin (Ramirez-Prado *et al.* 2018), or metabolic elicitors released to the medium (Munhoz *et al.* 2017; Wu *et al.* 2018).

The ability of the PGPR *P. fluorescens* N21.4 to trigger plant metabolism in different species has been described in numerous previous studies (Domenech *et al.* 2007; Ramos-Solano *et al.* 2010b; Algar *et al.* 2012; Ramos-Solano *et al.* 2015), and certain bacterial metabolic elicitors have been classified by their molecular weight (Algar *et al.* 2012). The present study explores further the complex mixture of elicitors produced by *P. fluorescens* based on solubility in three organic solvents.

The effectiveness of all three fractions to protect plants (Fig. 1) reveals the existence of several metabolic elicitors with contrasting solvent solubilities involved in plant protection. There may well be different pattern recognition receptors (PRRs) in plants for these elicitors.

Induced systemic resistance holds potential for activating cellular defence responses prior to pathogen attack (Akram *et al.* 2016). It is well known that, among others, ISR is accompanied with an augmented expression of defence-related genes, increased accumulation of secondary metabolites and defence-associated proteins (Conrath 2006; Zamioudis and Pieterse 2012). Moreover, the rapid generation of ROS is a common protective response of plants to pathogen attack (Noctor *et al.* 2014; Xia *et al.* 2015) and therefore represents the node from which many signalling events are generated.

Induced systemic resistance typically relies on JA/ET signalling pathways (Pieterse *et al.* 2002). Our results, at the level of gene expression and activity of proteins related to plant defence systems (Figs 2 and 3), indicate that elicitors from the *n*-hexane fraction induce the JA/ET pathway and also the SA pathway by increasing at the same time expression of marker genes *PR1* and *PDF1* (SA and JA/ET marker genes, respectively; Caarls *et al.* 2015; Ding *et al.* 2018) and enzyme activities such as PR2 (marker of SA signalling pathway) and PR3, LOX and PPO, as markers of JA/ET signalling pathway (Lucas *et al.* 2014; García-Cristobal *et al.* 2015; Silva *et al.* 2018; Wu *et al.* 2018).

This implies a versatility in the resistance mechanism, allowing attack of biotrophic and necrotrophic organisms to be opposed. These two pathways are not necessarily antagonistic, as previously been indicated by other results (Betsuyaku *et al.* 2017; Nie *et al.* 2017).

The physiological state induced by elicitors is known as priming. It is marked by an enhanced activation of defence mechanisms readily demonstrated in pathogen challenge experiments (Maunch-Mani *et al.* 2017). The induction of defensive mechanisms must necessarily be mediated by elicitor detection that activates an immune response. This has been termed microbe-associated molecular pattern (MAMP)-triggered immunity (MTI). It relies on the detection of conserved microbial signature molecules (MAMPs) via extracellular transmembrane receptors or PRRs (Mhlongo *et al.* 2018). Our results add to the picture by demonstrating a stimulation of all the ASC-GSH cycle enzyme of plants treated with *n*-hexane extracts from *P. fluorescens* (except, GR), notably for APX, GPX and MDHAR (Fig. 4). These enzymes have well-established roles in stress responses (Song *et al.* 2009; Sultana *et al.* 2012; García-Cristobal *et al.* 2015; Souza *et al.* 2016; Liu *et al.* 2018; Maruta and Ishikawa 2018).

The results obtained with respect to the enzymatic activities related to free-radical scavenging, accord with the suppressed levels of MDA (Fig. 5), a marker of oxidative damage (Lucas *et al.* 2017). These results are consistent with the higher protection and with the higher activity of the ROS scavenging enzymes reported above.

There are few studies that relate oxidative stress enzymes to innate immunity in plants elicited with PGPR or metabolic elicitors (Lucas *et al.* 2014; García-Cristobal *et al.* 2015). However, this type of relationship helps to stablish a complete set of changes associated to plant protection. Markers related to oxidative stress metabolism will assist in improving primer fingerprinting for each bacterial strain (Maunch-Mani *et al.* 2017; Gutierrez Albanchez *et al.* 2018). This will improve further analysis and also our understanding of the mechanisms that defend plants against pathogens. In addition, new sets of products based on metabolic elicitors or PGPR with an ability to elicit defence mechanisms against a range of stresses can be expected to be useful in practical agriculture.

## Conclusions

Extract from media in which *P. fluorescens* N21.4 was cultured using three different solvents each protected *A. thaliana* against the pathogen *P. syringae* DC3000, highlighting the *n*-hexane fraction. Extracts in *n*-hexane gave higher protection than those of ethyl acetate and butanol. The mode of action of the elicitors in the *n*-hexane fraction included activating SA, JA or ET signalling pathways and the enzymatic machinery of ROS scavenging to decrease oxidative stress. Further studies are needed to identify chemically the elicitors excreted by *P. fluorescens*. Once this is achieved, their use as biotechnological inoculants to improve the plant resistance to stress is a promising possibility.

## Supporting Information

The following additional information is available in the online version of this article—

**Table S1.** Primers forward and reverse used in qPCR analysis.

plz049_suppl_Supplementary_Information

## Sources of Funding

The authors would like to thank the Ministerio de Economía y Competitividad of Spain for funding this work through the project AGL-2013-45189-R.

## Conflict of interest

None declared.
