# Supplementary material for: Extracts from cultures of Pseudomonas fluorescens induce defensive patterns of gene expression and enzyme activity while depressing visible injury and reactive oxygen species in Arabidopsis thaliana challenged with pathogenic Pseudomonas syringae
Source: AoB Plants. 2019 Jul 29;11(5):plz049. doi: 10.1093/aobpla/plz049 (PMC6794073; doi:10.1093/aobpla/plz049)
Supplement: plz049_suppl_Supplementary_Information [file plz049_suppl_supplementary_information.docx]

**Table 1.** Primers forward and reverse used in qPCR analysis.

|  | **Forward** | **Reverse** |
| --- | --- | --- |
| *AtNPR1* | 5´-TATTGTCAARTCTRATGTAGAT-3´ | 5´-TATTGTCAARTCTRATGTAGAT-3´ |
| *AtPR1* | 5´-AGTTGTTTGGAGAAAGTCAG-3´ | 5´-GTTCACATAATTCCCACGA3´ |
| *AtICS* | 5´-GCAAGAATCATGTTCCTACC-3´ | 5´AATTATCCTGCTGTTACGAG-3´ |
| *AtPDF1* | 5´-TTGTTCTCTTTGCTGCTTTCGA-3´ | 5´-TTGGCTTCTCGCACAACTTCT-3´ |
| *AtLOX2* | 5´-ACTTGCTCGTCCGGTAATTGG-3´ | 5´-GTACGGCCTTGCCTGTGAATG-3´ |
| *AtMYC2* | 5´-GATGAGGAGGTGACGGATACGGAA-3´ | 5´-CGCTTTACCAGCTAATCCCGCA-3´ |
| *AtPR2* | 5´-TCGTCTCGATTATGCTCTCTTC-3´ | 5´-GCAGAATACACAGCATCCAAAA-3´ |
| *AtPR3* | 5´-AAATCAACCTAGCAGGCCACT-3´ | 5´-GAGGGAGAGGAACACCTTGACT-3´ |
| *Sand* | 5′ -CTGTCTTCTCATCTCTTGTC- 3′ | 5′ -TCTTGCAATATGGTTCCTG- 3′ |
